# CD147 Promotes Tumor Lymphangiogenesis in Melanoma via PROX-1

**DOI:** 10.3390/cancers13194859

**Published:** 2021-09-28

**Authors:** Coralie Reger de Moura, Alexandra Landras, Farah Khayati, Uwe Maskos, Kamel Maouche, Maxime Battistella, Suzanne Menashi, Céleste Lebbé, Samia Mourah

**Affiliations:** 1 Inserm, UMR_S976, Université de Paris, 75010 Paris, France; coralie.reger@aphp.fr (C.R.d.M.); alexandra.landras@inserm.fr (A.L.); farah.khayati@inserm.fr (F.K.); maxime.battistella@aphp.fr (M.B.); celeste.lebbe@aphp.fr (C.L.); 2 Pharmacogenomics Department, Saint Louis Hospital, AP-HP, 75010 Paris, France; menashi@u-pec.fr; 3 Neurobiologie Intégrative des Systèmes Cholinergiques, Institut Pasteur, Université de Paris, CNRS, UMR_3571, 75724 Paris, France; uwe.maskos@pasteur.fr; 4 Biology and Pathology of the Endocrine Pancreas, Université de Paris, CNRS, UMR_8251, 75013 Paris, France; kamel.maouche@univ-paris-diderot.fr; 5 Pathology Department, Saint Louis Hospital, AP-HP, 75010 Paris, France; 6 Dermatology Department, Saint Louis Hospital, AP-HP, 75010 Paris, France

**Keywords:** lymphangiogenesis, CD147, melanoma, lymph node metastasis, therapeutic targets

## Abstract

**Simple Summary:**

Melanoma is one of the most aggressive skin cancers, characterized by metastasis to the lymph nodes and a high capacity to develop drug resistance. There is a lack of knowledge on the mechanisms contributing to lymphatic vessel formation and metastasis regulation in malignant melanoma. We previously reported the involvement of CD147, a transmembrane glycoprotein overexpressed in melanoma, in the regulation of the tumor microenvironment and angiogenesis. The aim of our study was to further determine how CD147 is involved in lymphangiogenesis regulation. Our results revealed that high CD147 expression is correlated with the number of lymphatic vessels in the human melanoma lymph nodes and that paracrine CD147 upregulates lymphangiogenesis through lymphangiogenic mediators in vitro and in vivo, suggesting that CD147 could be a promising target for melanoma-associated lymphangiogenesis inhibition.

**Abstract:**

Malignant melanoma is one of the most aggressive skin cancers and is characterized by early lymph node metastasis and the capacity to develop resistance to therapies. Hence, understanding the regulation of lymphangiogenesis through mechanisms contributing to lymphatic vessel formation represents a treatment strategy for metastatic cancer. We have previously shown that CD147, a transmembrane glycoprotein overexpressed in melanoma, regulates the angiogenic process in endothelial cells. In this study, we show a correlation between high CD147 expression levels and the number of lymphatic vessels expressing LYVE-1, Podoplanin, and VEGFR-3 in human melanoma lymph nodes. CD147 upregulates in vitro lymphangiogenesis and its related mediators through the PROX-1 transcription factor. In vivo studies in a melanoma model confirmed that CD147 is involved in metastasis through a similar mechanism as in vitro. This study, demonstrating the paracrine role of CD147 in the lymphangiogenesis process, suggests that CD147 could be a promising target for the inhibition of melanoma-associated lymphangiogenesis.

## 1. Introduction

Lymphangiogenesis is widely exploited by cancer cells in order to disseminate and form metastases [1]. Lymph node metastasis, lymphatic vessels’ density, and lymphangiogenic growth factor levels are known to be associated with poor prognosis and aggressive potential in solid tumors, including melanoma [2]. Several lymphangiogenic regulators are involved in the metastatic potential, among them the vascular endothelial growth factor receptors VEGFR-3 and VEGFR-2 and their ligands VEGF-C and VEGF-D, which promote lymphatic endothelial cell (LEC) proliferation and lymph node metastasis formation [3,4,5], as well as LYVE-1 and Podoplanin [6]. LYVE-1 is a specific marker of lymphatic vessels that binds, internalizes, and thus transports hyaluronan from the tissue to the lymph [7]. Podoplanin is another selective marker of the lymphatic endothelium associated with tumor lymphangiogenesis and metastasis, although it is also expressed by other cell types [8,9]. These lymphangiogenic markers are regulated by the transcription factor PROX-1, which is a key player in LECs differentiation and thus regulates lymphatic system development [2,10,11].

In human metastatic melanoma, the expression of LYVE-1, Podoplanin, and VEGFR-3 is associated with lymphatic invasion, lymph node metastasis, and poor patient survival [12,13,14,15,16,17]. Moreover, tumor lymphangiogenesis is induced in melanoma through the secretion of these lymphangiogenic growth factors, leading to disease progression. Indeed, several studies aimed to improve predictive biomarkers in order to detect melanoma metastasis [18,19].

Our previous works have shown that the membrane glycoprotein CD147 regulates the tumor microenvironment and angiogenesis in a paracrine manner [20,21]. Several studies have shown that CD147 is overexpressed in more than 20 different types of cancers and that its level of expression is correlated with poor prognosis, including reduced overall survival rates [22]. Moreover, our study on a large series of primary and metastatic melanomas showed that CD147 overexpression was associated with reduced overall survival in patient with primary tumors, and was also correlated with metastasis and vertical invasion, demonstrating that CD147 is an important prognostic biomarker involved in melanoma’s aggressiveness [23]. A retrospective study reported a correlation between CD147 overexpression and malignant phenotype, such as lymph node invasion and metastasis, in non-small-cell lung cancer [24]. More recently, the regulation of invasion and lymph node metastasis through lipid metabolism, controlled by CD147 expression in cervical cancer, has been demonstrated, suggesting that CD147 promotes tumor lymphangiogenesis through fatty acid synthesis [25].

Thus, lymphangiogenesis represents an essential treatment target and it is important to understand the regulation of signaling pathways in lymphatic endothelial cells and in lymphatic vessel development associated with metastatic melanoma. In this study, we set out to investigate the potential role of CD147 in the lymphangiogenic process and its consequences for melanoma metastasis.

## 2. Materials and Methods

### 2.1. Patients and Specimens

The research with metastatic melanoma specimens, collected from 16 patients who underwent surgery between January 2007 and December 2010 in the Department of Dermatology, was approved by the Ethics Committee of Saint Louis Hospital (IRB N° 00006477). All patients provided written informed consent. All cases diagnosed were reviewed by the Department of Pathology. Patient follow-up and surgical excision of melanoma were performed according to the 2009 AJCC recommendations. The review in standardized forms of clinical records was achieved. The clinical data are summarized in Table S1.

### 2.2. Immunohistochemistry

Immunohistochemical staining was performed in formalin-fixed, paraffin-embedded (FFPE) tissue sections. It was performed by indirect manual immunostaining using the avidin–biotin–peroxidase method. All sections were deparaffinized in xylene and dehydrated through a graduated alcohol series. Antigen retrieval was performed in a citrate buffer with pH 6 at 95 °C for 20 min. To prevent endogenous peroxidase activity, the slides were treated with 3% peroxidase (H_2_O_2_) for 15 min and blocked with Horse serum from a Vectastain ABC kit (Vector Laboratories, Burlingame, CA, USA). The slides were then incubated with Mouse anti-CD147 monoclonal antibody (#555961, BD Pharmingen, San Jose, CA, USA) in 0.5% PBS-BSA, overnight at 4 °C. According to the manufacturer’s protocol, the biotinylated secondary antibody was incubated for 30 min at room temperature and detected with a streptavidin–peroxidase complex. Then, peroxidase activity was detected using the AEC method for 10 min. Sections were counterstained with hematoxylin, rehydrated through graduated alcohol series, and mounted with a xylene mounting medium (Eukitt, Dutscher, Bernolsheim, Belgium).

### 2.3. Immunofluorescence Staining

Four micrometer-thick frozen tumor sections or LECs, previously treated or not with 5 µg/mL of recombinant human CD147 (rhCD147) (R&D Systems, Minneapolis, MN, USA) for 30 min, were fixed in 4% formaldehyde for 20 min and permeabilized with 0.1% Triton X-100 in PBS for 10 min. Tumor sections, or LECs, were then incubated with a blocking solution of PBS containing 5% of BSA and 0.05% Tween20. Sections or cells were then incubated with Rabbit anti-LYVE-1 polyclonal antibody (#ab14917, Abcam, Cambridge, UK), Rabbit anti-VEGFR-3 polyclonal antibody (#102-PA22AG, ReliaTech, Wolfenbuttel, Germany), Rabbit anti-PROX-1 polyclonal antibody (#07-537, Merck, Darmstadt, Germany) or Mouse anti-Podoplanin monoclonal antibody (#ab10288, Abcam) overnight at 4 °C, washed three times with PBS-0.05% Tween20, and labeled with a Goat anti-Rabbit IgG (H+L) Cross-Absorbed Secondary Antibody Alexa Fluor 488 conjugate (#A11008, Thermo Fisher Scientific, Waltham, MA, USA) or a Goat anti-Mouse IgG (H+L) Cross-Absorbed Secondary Antibody Alexa Fluor 488 conjugate (#A21121, Thermo Fisher Scientific) for 1 h. Samples were washed three times with PBS-0.05% Tween20, and then coverslips were mounted with Vectashield Mounting Medium with Dapi (Vector Laboratories, Burlingame, CA, USA). Slides were observed with a Confocal LSM800 (Zeiss LSM 800, Oberkochen, Germany).

A human melanoma lymph node staining study was performed in FFPE tissue sections. All sections were deparaffinized in xylene and dehydrated through a graduated alcohol series. Immunofluorescence staining was then performed as already described.

### 2.4. Cell Culture

Human BLM melanoma cells (ATCC, Manassas, VA, USA), chosen for their high metastatic potential [26,27], were stably transfected with scrambled microRNA or CD147-microRNA [20], were maintained in DMEM containing 4.5 g/L glucose, supplemented with 10% fetal bovine serum (FBS), 2 mML-glutamine, 100 U/mL penicillin, and 100 mg/mL streptomycin (Gibco, Thermo Fisher Scientific, MA, USA).

Human dermal lymphatic endothelial cells (HDLECs) (Promocell, Heildelberg, Germany) were maintained in complete Endothelial Cell Growth Medium MV2 according to the manufacturer’s instructions. The cells were passaged at 70% confluence with a suitable DetachKit (Promocell, Heidelberg, Germany).

Chinese hamster ovary (CHO) cells (ATCC, Rockville, MD, USA) were maintained in DMEM/F12 (Invitrogen, Thermo Fisher Scientific, MA, USA), supplemented with 10% FBS and 2 mML-glutamine. CHO cells were transfected with a plasmid containing CD147 full-length cDNA or not, as previously described [28]. Stably transfected cells were designated CHO-CD147 cells or CHO-Empty Vector cells. CHO-CD147 and CHO-Empty Vector membrane vesicles were isolated by differential centrifugation, as previously described [21,29].

### 2.5. In Vivo Monitoring Assays

Animals were handled under stringent sterile and controlled conditions (temperature, humidity, and light cycle (12 h/12 h)). Immunodeficient nude mice (NMRI-Foxn1 nu/nu) are a common model used in subcutaneous xenograft procedures. All protocols were approved by the Committee on the Ethics of Animal Experiments of the French Ministry of Agriculture (Permit Number: B75-10-2014). Moreover, methods were performed in accordance with the relevant guidelines and regulations in accordance with Directive 2010/63/EU. All studies involving animals are reported in accordance with the ARRIVE guidelines for reporting experiments. The rules of replacement, refinement, and reduction (“the 3 Rs”) were respected in order to reduce the suffering of the animals and the number of animals.

Female five-week-old NMRI-Foxn1 nu/nu mice (16–20 g) (Janvier Labs, Le Genest-Saint-Isle, France) were injected subcutaneously with 5 × 10^6^ stably transfected BLM-scrambled-miRNA or BLM-CD147-miRNA cells (*n* = 10 mice per condition). Tumor growth was monitored every 2–3 days, independently by two technicians with a digital caliper. Tumor volume was calculated using the formula: Length × Width^2^/2, and mice were euthanized by cervical dislocation when it reached a volume of approximately 2000 mm^3^. Resected tumors were immediately frozen and stored at −80 °C before cryostat sectioning. Liver, lung, and lymph nodes were fixed in 4% formalin and embedded in paraffin. Statistical analyses were performed using GraphPad Prism5 software (San Diego, CA, USA).

### 2.6. Metastasis Foci Counting

Tissue sections of 5 µm of FFPE liver, lung, and lymph nodes were taken. Metastatic foci were found on serial sections, every 300 µm, at different levels in each type of organ. All sections were deparaffinized in xylene and analyzed under confocal fluorescent microscopy. The number of metastatic foci was determined in a blinded manner by two independent technicians. The metastatic index was calculated using the formula: (metastasis foci)/(mean tumor size) × 10^3^.

### 2.7. In Vitro Migration, Proliferation, and Apoptosis Assays

In vitro migration assays were performed using a modified Boyden chamber fitted with 8 µm pore filter inserts (BD Bioscience, San Jose, CA, USA), and placed in 24-well plates. LECs were seeded, at a density of 1.5 × 10^3^ cells/insert, on the upper side of the chamber in a serum-free medium containing a mix of VEGF-A, VEGF-C, and bFGF (R&D Systems) at 100 ng/mL as a positive control or rhCD147 at 5 µg/mL. The lower chamber was filled with media supplemented with 1% FBS. After 24 h of incubation at 37 °C, the cells were fixed and stained with a solution of 0.5% crystal violet. The cells present in the whole lower side of the filter were counted under a light microscope.

In vitro cell proliferation and apoptosis assays were conducted with LECs treated with either a positive control mix at 100 ng/mL, rhCD147 at 5 µg/mL, or a serum-free medium. Briefly, cells were seeded in a 96-well plate at a density of 3000 cells/well in a medium supplemented with 10% FBS. After cell attachment, a positive control mix or rhCD147 in serum-free media was added to the LECs (100 µL/well) in triplicate. Cell proliferation was measured using the CellTiter-Glo^®^ assay (Promega, Charbonnières, France) 72 h after seeding, as described by the manufacturer. All conditions were evaluated in triplicate. Apoptosis was evaluated by measuring the level of Caspase 3/7 activity using the Caspase-Glo^®^ 3/7 Assay System (Promega, Charbonnières, France), as described by the manufacturer.

### 2.8. Tube Formation Assay

In vitro tube formation assay was performed on six-well plates precoated with human fibrinogen (VWR, Fontenay-sous-Bois, France) and human thrombin (Calbiochem, Merck) and suspended in PBS. After overnight equilibration with a MV2 medium supplemented with 2% FBS, LECs were seeded, at a density of 2.5 × 10^5^ cells/well, in serum-free media alone or supplemented with a positive control mix at 100 ng/mL, or with rhCD147 at 5 µg/mL. The formation of a tube-like structure was photographed with a light microscope 24 h after cell addition. As a quantitative estimation of lymphangiogenesis, the tube length obtained from four different fields were carried out using ImageJ software (http://imagej.nih.gov/ij/, Bethesda, MD, USA).

### 2.9. Plasmids and siRNA Transfection

CD147 stable knockdown of microRNA was performed as previously described [20].

Two siRNAs for PROX1 (ID: s11228: 3′GUUUGAUAUGGAUCGCUUAtt5′ and 5′ UAAGCGAUCCAUAUCAAACtg3′ and ID: s11227: 3′ CCUGAAUCCUUAGACUUAAtt and 5′ UUAAGUCUAAGGAUUCAGGag3′) or scrambled siRNA oligos (Ambion, Thermo Fisher Scientific, MA, USA) were transfected into LECs using Lipofectamine-2000 (Invitrogen, Thermo Fisher Scientific MA, USA), as described by the manufacturer. After 4 h in serum-free media, the transfected cells were incubated in complete MV2 media for 24 h, and then analyzed for lymphangiogenesis markers.

### 2.10. Western Blot Analyses

LECs were seeded on six-well plates, at a density of 2.5 × 10^5^ cells/well, and treated or not with rhCD147 at 5 µg/mL for 1 h. Next, cells were washed with cold PBS and then lysed in RIPA buffer (Thermo Fisher Scientific, MA, USA) supplemented with Phosphatases and Proteases Inhibitor Cocktail (Roche, Bâle, Switzerland) on ice. After 1 h on ice, lysates were centrifuged at 13,000 g for 20 min at 4 °C. The protein concentration was determined using BCA kit (Pierce, Thermo Fisher Scientific, MA, USA). Then, 20 µg of lysate in 4× Laemmli per lane were resolved by reducing 10% SDS-PAGE followed by Western blot analyses with either Rabbit anti-LYVE-1 polyclonal antibody (#ab14917, Abcam, Cambridge, UK), Rabbit anti-VEGFR-3 polyclonal antibody (#102-PA22AG, ReliaTech), Rabbit anti-PROX-1 polyclonal antibody (#07-537, Merck), Mouse anti-Podoplanin monoclonal antibody (#ab10288, Abcam, Cambridge, UK) or Mouse monoclonal anti-β-actin antibody (#A2228, Merck) for loading control. The antigen–antibody complexes were visualized with ECL reagent (Pierce, Thermo Fisher Scientific, MA, USA). Their fold change expression was determined by densitometry using ImageJ software (http://imagej.nih.gov/ij/, Bethesda, MD, USA). All the uncropped Western Blot images were included in Figure S1.

### 2.11. Real-Time Quantitative PCR (qRT-PCR)

Total RNA was extracted from LECs using a Maxwell RSC simplyRNA tissue kit (Promega) according to the manufacturer’s protocol. The quality and quantity of mRNA were determined using a NanoDrop ND-1000 spectrophotometer (NanoDrop Technologies, Thermo Fisher Scientific). First-strand cDNA was synthesized from 1 µg of total RNA using a High-Capacity cDNA Archive Kit (Applied Biosystems, Thermo Fisher Scientific) according to the manufacturer’s protocol. The obtained cDNA samples were diluted four times for quantitative qPCR assay. *LYVE1*, *VEGFR3*, *PROX1*, and *PDPN* primers were specifically designed (Eurogentec, Liège, Belgium and Anygenes, Paris, France). Transcript levels were measured by qRT-PCR using Perfect Master Mix-Probe (Roche) on LightCycler-480 (Roche), according to the manufacturer’s protocol, and were normalized to the housekeeping Peptidylprolyl isomerase A (*PPIA*) gene transcripts.

### 2.12. Chromatin Immunoprecipitation (ChIP)

Serum-starved LECs, treated or not with rhCD147 at 5 µg/mL for 4 h, were fixed with 4% formaldehyde (Merck), lysed and subject to ChIP assay (Merck), according to the manufacturer’s protocol. Fragments of PROX1 and chromatin complexes were immunoprecipitated with a Rabbit anti-PROX-1 polyclonal antibody (#07-537, Merck). Negative and positive controls from a ChIP assay kit were used. DNA fragments bound to *PROX1* were subjected to a qPCR analysis. Primers for the *PROX1* promoter-binding site were specifically designed by Anygenes (Paris, France); the information is listed in Table S2.

### 2.13. Human Phosphokinase Array

LECs were seeded on six-well plates, at a density of 2.5 × 10^5^ cells/well, and treated or not with rhCD147 at 5 µg/mL for 5 min. Cell lysates were incubated with the phosphokinase arrays (#ARY003, R&D Systems) overnight at 4 °C, according to the manufacturer’s protocol. Signals were visualized with ECL reagent (Pierce, Thermo Fisher Scientific, MA, USA). The spot signals were quantified using ImageJ software.

### 2.14. Statistical Analysis

Data are expressed as mean ± SD. The statistical significance of a difference between two groups was determined by an unpaired Student’s *t*-test. One-way analysis of variance (ANOVA) was used to compare differences between groups for siRNA-PROX-1 experiments. Differences were considered significant when *p* < 0.05. GraphPad Prism 5 was used for the statistical analyses.

## 3. Results

### 3.1. CD147 Expression Is Associated with Lymphangiogenesis Mediators in Human Melanoma Lymph Node Metastasis

**Figure 1:**
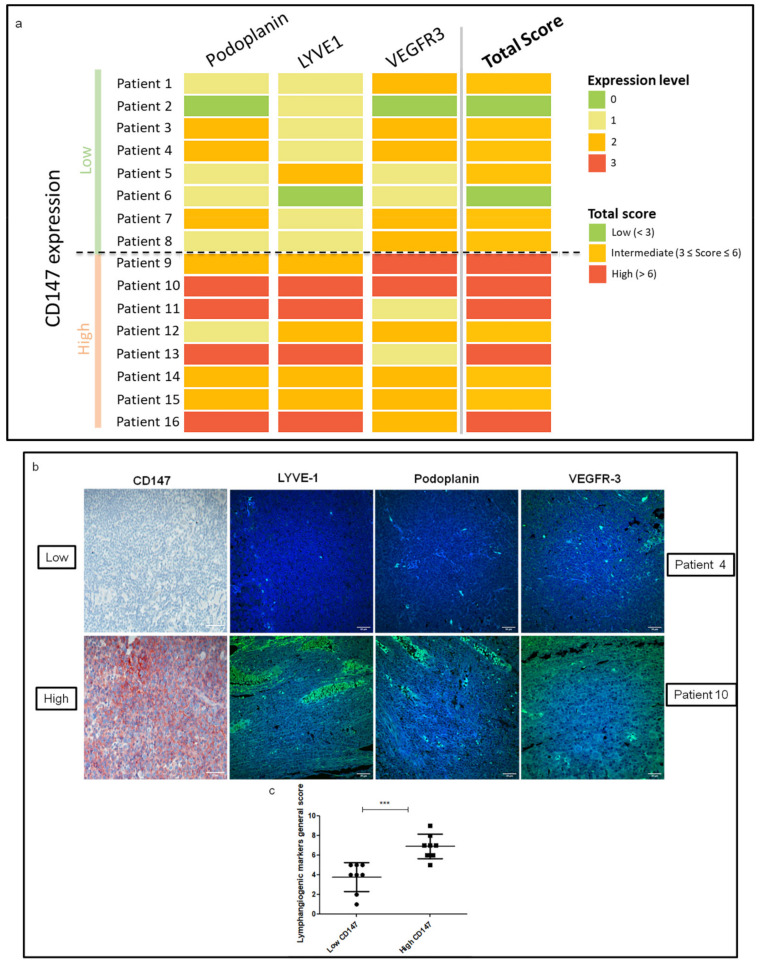
Lymphangiogenic markers’ expression in human melanoma lymph nodes expressing high or low CD147. (**a**) Level of expression of Podoplanin, LYVE-1, and VEGFR-3 according to CD147 expression in human melanoma lymph nodes. The intensity of staining was scored between 0 and 3 for each marker and CD147, and the total lymphangiogenic score was classified as high, intermediate, or low. (**b**) Representative images of CD147 immunohistochemistry staining and LYVE-1, Podoplanin, and VEGFR-3 immunofluorescence staining in melanoma lymph node samples of patient 4 (low CD147 expression) and patient 10 (high CD147 expression) (40× magnification). Scale bar: 30 µm. (**c**) Vertical scatter plot with mean of lymphangiogenic markers general scores of low vs. high CD147 expression in human melanoma lymph nodes. SD; *** *p* < 0.0001.

An immunofluorescence study was performed to evaluate the expression of the three main lymphangiogenesis markers, Podoplanin, LYVE-1 and VEGFR-3, on 16 resected human melanoma lymph node metastases harboring high or low CD147 expression (Table S1). Figure 1a shows an expression profile where the higher the lymphangiogenesis markers’ expression in the melanoma lymph nodes, the higher the expression of CD147. Immunohistochemical analysis also showed that the level of CD147 staining had the same trend as the intensity of the immunofluorescence of the lymphatic vessel markers LYVE-1 and Podoplanin. Moreover, intratumoral VEGFR-3 staining was more intense in melanoma lymph nodes containing high amounts of CD147 (Figure 1b,c).

### 3.2. CD147 Promotes In Vitro Lymphangiogenesis

Since tumor cells overexpress CD147 on their cell membrane, which can then interact with the lymphatic cells, we sought to evaluate the potential paracrine effect of CD147 on lymphendothelial primary cells’ (LECs) properties, including cell migration, proliferation, survival, and tubulogenesis, using a recombinant human CD147 (rhCD147).

**Figure 2:**
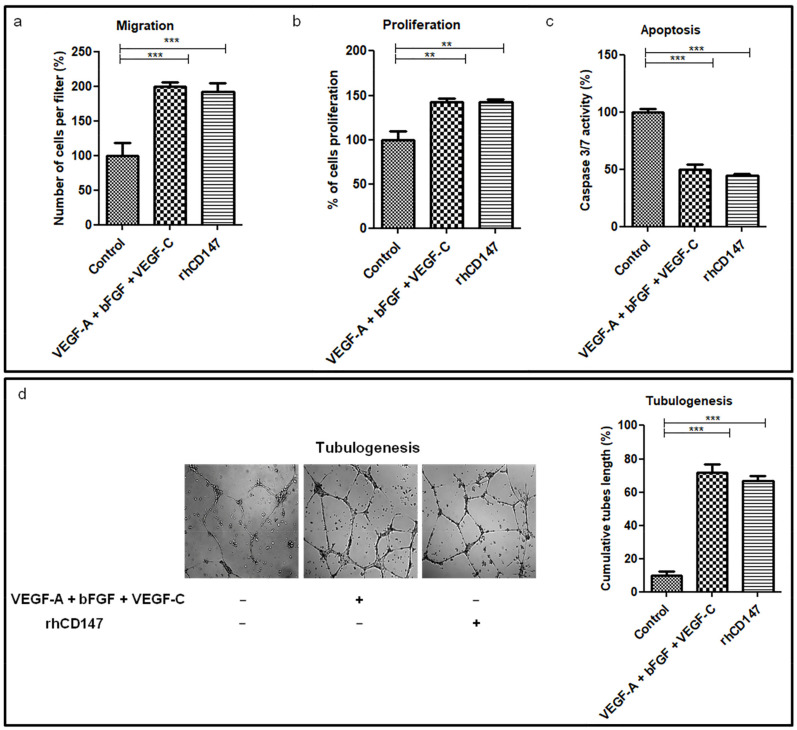
CD147 modulates lymphangiogenic properties of LECs. LECs were treated with or without rhCD147, or with a positive control mix (VEGF-A + bFGF + VEGF-C) and evaluated for effects on cell migration (**a**), proliferation (**b**), apoptosis (**c**), and tubulogenesis (**d**). Columns indicate the means of three independent experiments carried out in triplicate and bars indicate the SD; ** *p* < 0.005, *** *p* < 0.0001.

As shown in Figure 2a, rhCD147-treated LECs displayed an increase of 92% (mean +/−11%) in their ability to migrate through Matrigel-coated filters compared to untreated cells. Moreover, rhCD147 significantly increased LECs’ proliferation by 43% (mean +/−4%) (Figure 2b), while apoptosis was significantly reduced by 55% (mean +/−2%), as determined by Caspase 3/7 activity (Figure 2c). Next, we examined the effect of rhCD147 on capillary-like tube formation in LECs by measuring the capillary tube length. As shown in Figure 2d, rhCD147 stimulated tubulogenesis by approximately seven-fold compared to untreated LECs.

As both soluble and membrane-vesicle-bound CD147 have been suggested to modulate the tumor–stromal interaction and act as paracrine modulators, we also performed additional experiments with CD147-enriched membrane vesicles from CD147 full-length cDNA CHO-transfected cells [21,28]. As shown in Figure S2, CHO-CD147 also significantly increased LECs’ proliferation and migration.

Overall, these results show that rhCD147 enhances significantly in vitro lymphangiogenesis.

### 3.3. CD147 Regulates Lymphangiogenesis Mediators through PROX-1 Transcription Factor

**Figure 3:**
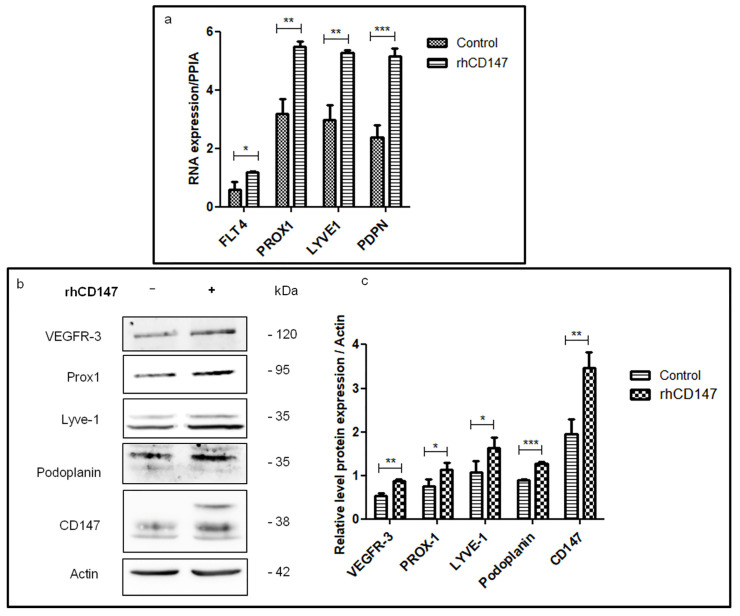

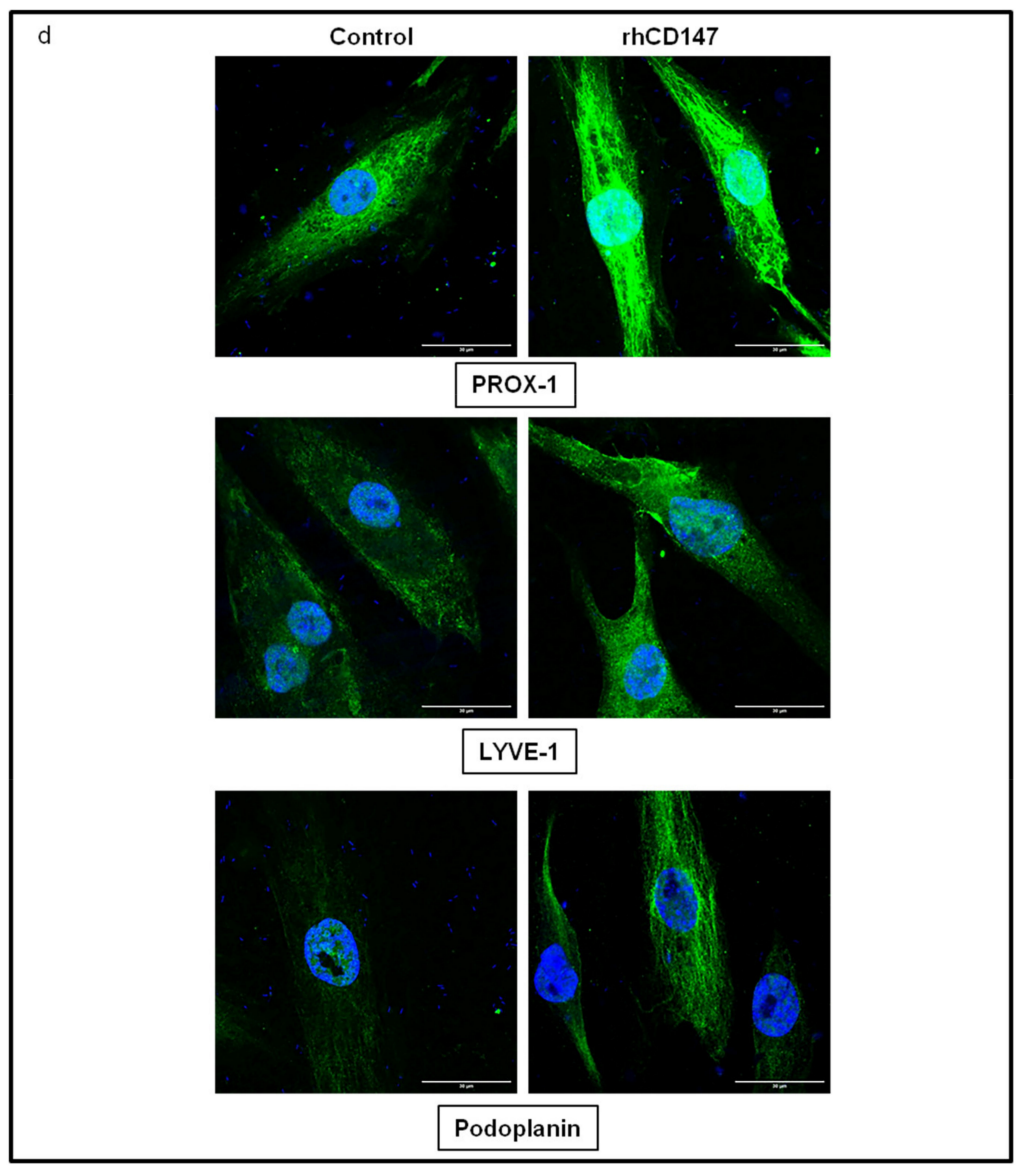

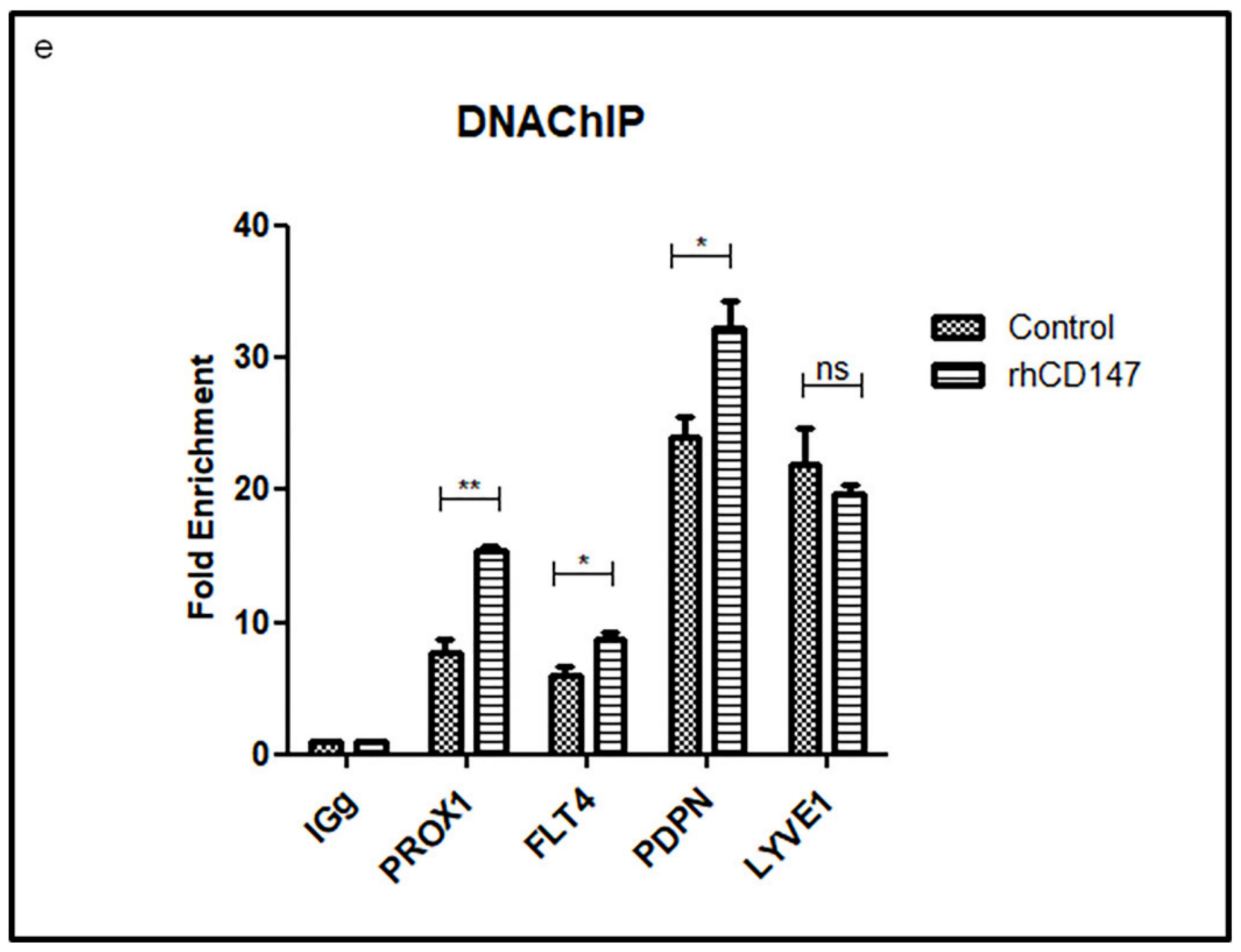
CD147 regulates LECs lymphangiogenic markers at transcriptional and translational levels. (**a**) qRT-PCR of *FLT4*, *PROX1*, *LYVE1*, and *PDPN* expression in LECs treated or not with rhCD147, using *PPIA* as a reference. Columns indicate the means of three independent experiments carried out in triplicate; bars indicate SD; * *p* < 0.05, ** *p* < 0.005, *** *p* < 0.0001. (**b**) Western blot analyses of VEGFR-3, PROX-1, LYVE-1, Podoplanin, and CD147 expression in total lysates of LECs treated or not with rhCD147. (**c**) Three independent Western blots were quantified for densitometry band analyses. Equal loading of proteins was assessed by probing for β-actin. Bars, SD; * *p* < 0.05, ** *p* <0.005, *** *p* < 0.0001. (**d**) Representative confocal images of PROX-1, LYVE-1 and Podoplanin immunofluorescent staining on LECs treated or not with rhCD147 (63X magnification). Scale bar: 30 µm. (**e**) ChIP-qPCR was performed on LECs treated or not with rhCD147, with antibody against PROX-1 total and control IgG. Columns indicate means of two independent experiments; and bars, SD; ns, non significant, * *p* < 0.05, ** *p* < 0.005, *** *p* < 0.0001.

We then examined whether the in vitro functional effects induced by rhCD147 on lymphangiogenesis are supported by potential regulation of lymphangiogenic-specific mediators, including VEGFR-3 (the main tyrosine kinase receptor involved in lymphangiogenesis [30]), PROX-1 (a transcription factor known to be involved in lymphatic endothelial differentiation [11]), Podoplanin (a selective marker of lymphatic endothelium [31]), and LYVE-1 (a lymphatic endothelial specific receptor [7]). For that, we first examined by RT-qPCR the transcript expression profile of these factors in LECs treated or not with rhCD147 (the positive control mix (VEGF-A + bFGF + VEGF-C) is shown in Figure S3). As shown in Figure 3a, *VEGFR3*, *PROX1*, *LYVE1*, and *PDPN* transcripts were upregulated by 100%, 72%, 77%, and 116% (mean +/−0.02, 0.2, 0.1, and 0.25), respectively, under rhCD147 treatment. Immunoblotting analyses confirmed this upregulation at a translational level. Indeed, LECs ‘treatment with rhCD147 was associated with an increase in the expression of VEGFR-3, PROX-1, LYVE-1, and Podoplanin by 38%, 32%, 34%, and 29%, respectively (Figure 3b,c). The immunoblotting results were confirmed by immunofluorescence analyses since immunostaining revealed a clear upregulation of these lymphangiogenic factors (Figure 3d). Interestingly, PROX-1 immunostaining also showed an increase in nucleus localization and translocation.

Next, using a ChIP assay coupled to qPCR analyses, we examined whether the transcriptional factor PROX-1 mediates the observed effect of rhCD147 in enhancing lymphatic endothelial markers’ expression. As shown in Figure 3e, rhCD147 treatment of LECs significantly increased the binding of PROX-1 to different promoters of lymphangiogenic markers. Indeed, *PROX1*, *FLT4*, and *PDPN* promoter regions containing PROX-1 binding sites were increased by 2-, 1.4-, and 1.3-fold, respectively, under treatment. Thus, rhCD147 treatment regulates PROX-1 recruitment to lymphangiogenic targets’ promoters. However, rhCD147 did not show any effect on *LYVE1* promoter regions containing a PROX-1 binding site, suggesting an additional mechanism could be involved in LYVE-1 regulation by CD147.

**Figure 4:**
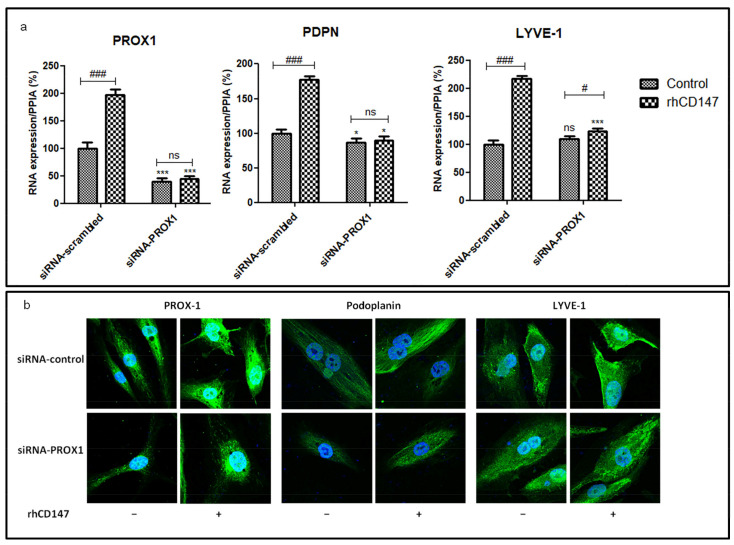
CD147 regulates LECs lymphangiogenic markers through PROX-1 transcription factor. LECs were transfected with scrambled or PROX-1 siRNA and treated or not with rhCD147. (**a**) qRT-PCR of *PROX1*, *PDPN*, and *LYVE1* expression using *PPIA* as a reference. Columns indicate means of three independent experiments carried out in triplicate; bars indicate SD; * *p* < 0.05, *** *p* < 0.0001 for siRNA-PROX-1 vs. siRNA-scrambled. ns, nonsignificant, # *p* < 0.05, ### *p* < 0.0001 for treated vs. untreated cells. (**b**) Representative confocal images of immunofluorescence staining for PROX-1, Podoplanin and LYVE-1 (63× magnification).

Furthermore, the downregulation of PROX-1 using siRNA transient transfection (60% decrease compared to siRNA-scrambled) in LECs treated or not with rhCD147 confirmed the impact of rhCD147 on the lymphangiogenic markers. Indeed, *PDPN* mRNA expressions decreased, albeit only partially, after PROX-1 downregulation, with a significant decrease of 23% compared to siRNA-scrambled. Interestingly, *LYVE1* was not regulated by PROX-1 downregulation. As expected, rhCD147 treatment induced *PROX1*, *PDPN*, and *LYVE1* expression in scrambled transfected cells (means: 96%, 77%, and 117%, respectively). Importantly, PROX-1 siRNA-treated LEC cells no longer responded to rhCD147 treatment without regulation of *PROX1*, *LYVE1*, and *PDPN*, confirming that PROX-1 is involved in the CD147 modulation of these lymphangiogenic markers (Figure 4a). These results were confirmed by an immunofluorescence analysis (Figure 4b). Collectively, these results suggest that lymphangiogenic factors’ regulation by CD147 is in part mediated by PROX-1.

**Figure 5:**
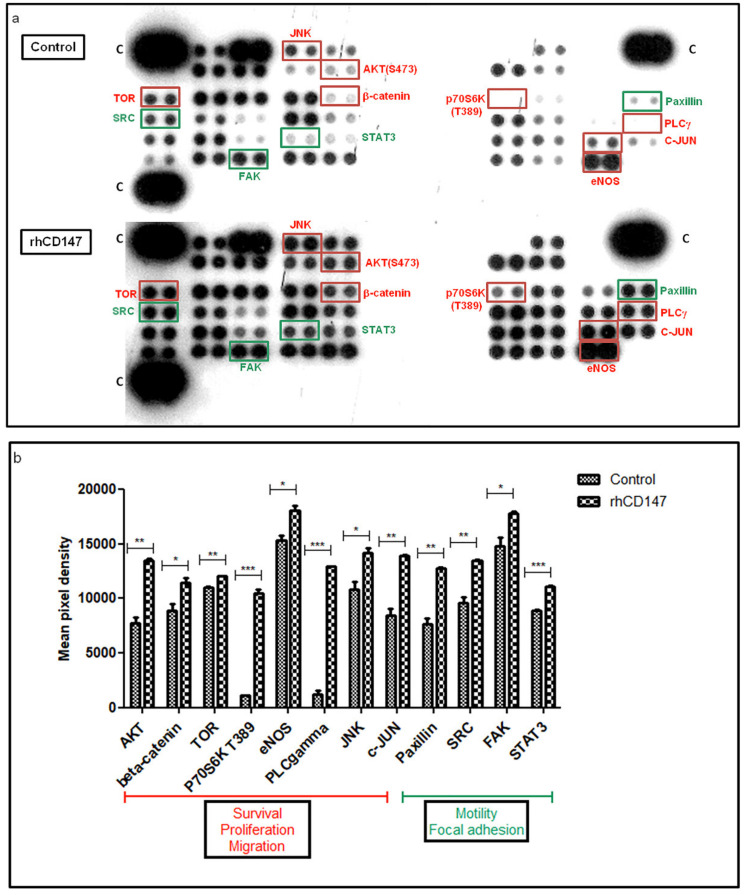
Signaling pathways involved in CD147 lymphangiogenesis regulation. (**a**) Whole lysates from LECs treated or not with rhCD147 were collected for human phosphokinase array analysis. Each membrane contains 46 specific kinases and a positive control (C). Antibodies were spotted in duplicate. (**b**) Mean pixel density of spots was quantified. Each bar represents the mean of duplicate spots. Bars represent SD; * *p* < 0.05, ** *p* < 0.005, *** *p* < 0.0001.

Next, we examined the signaling pathways involved in CD147-regulated lymphangiogenesis, using a phosphoprotein array including 46 kinase phosphorylation sites and their known effector proteins. The relative phosphorylation levels of several proteins involved in lymphangiogenesis were significantly higher in the rhCD147-treated LECs (Figure 5a,b). Indeed, known signaling pathways downstream of VEGFR-3 were markedly activated, such as PI3K/AKT/mTOR/P70S6K [32], AKT/eNOS [33], AKT/β-catenin [32], and AKT/PLCγ [4], which are known to promote cell survival, migration, and proliferation [4,32,33]. Moreover, the SRC/FAK/Paxillin/STAT3 pathway, which regulates cell motility and focal adhesion complex formation [34,35,36], and the JNK/c-JUN signaling pathway were significantly activated in the rhCD147-treated LECs. This latter pathway is known to contribute to proliferation and apoptosis regulation [37], and, more recently, its significance for lymphatic vessel formation and LEC migration has been shown [38]. These results suggest that the signal transduction cascades involved in lymphangiogenic factors are regulated by CD147.

### 3.4. CD147 Downregulation Decreases Metastasis Formation and Lymphangiogenic Factors in a Human Melanoma Model

**Figure 6:**
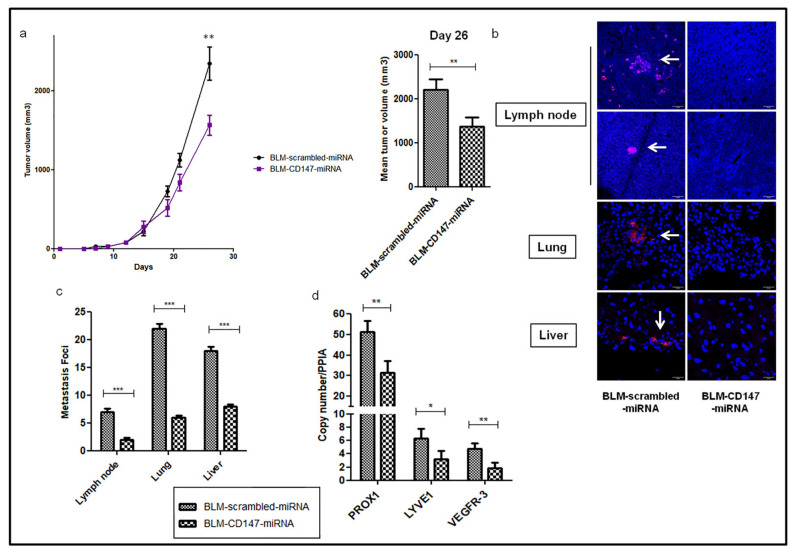
CD147 downregulation reduces tumor growth and metastasis formation in human melanoma. (**a**) In vivo xenograft growth. BLM-scrambled and BLM-CD147-miRNAs were inoculated in nude mice for 26 days. Results represent two independent experiments with 10 mice in each group. Columns represent means of tumor volume at sacrifice. Bars; SEM, ** *p* < 0.005. (**b**) Representative images of confocal fluorescent micro-metastasis (white arrows) in mice’s lymph node, lung and liver. 40x Magnification. Scale bar: 20 µm. (**c**) Quantification of the micro-metastatic foci in mice lymph nodes, lungs, and livers. (**d**) qRT-PCR of *PROX1*, *LYVE-1*, and *VEGFR3* expression in BLM-scrambled or BLM-CD147-miRNAs tumors, using *PPIA* as a reference. Columns indicate means of three independent experiments carried out in triplicate; and bars, SD; * *p* < 0.05, ** *p* < 0.005, *** *p* < 0.0001.

To investigate the effect of CD147 in the formation of metastases in melanoma, a stable knockdown of microRNA-targeting CD147, expressing mCherry fluorescent protein, was established in a BLM melanoma cell line (BLM-CD147-miRNA) [20]. The BLM cell line, isolated from a lung metastasis from the BRO cell line [39], is known to have high metastatic potential in nude mice, specifically in the lungs after approximately 40 days of cell inoculation [26,27,40], but also in other organs such as the brain [41]. Scrambled and CD147-miRNA BLM cells were inoculated into nude mice, and tumor growth and metastases development were examined after one month of the xenograft. As shown in Figure 6a, downregulation of CD147-miRNA reduced tumor growth, with a significant mean reduction in tumor size at sacrifice of 38%. Moreover, the number of metastasis foci in the lymph nodes, lungs, and liver detected using fluorescent microscopy showed significant decreases of 71%, 73%, and 56%, respectively, in BLM-CD147-miRNA compared to scrambled-miRNA injected mice (Figure 6b,c). The metastatic index suggests that the inhibition of CD147 has a greater effect on the metastasis formation as compared to tumor growth (Figure S4).

Next, we examined, by RT-qPCR, the transcript expression profile of the lymphangiogenic mediators in resected mice tumors. As shown in Figure 6d, *PROX-1*, *LYVE-1*, and *VEGFR-3* were downregulated by 39%, 51%, and 62%, respectively, in BLM-CD147-miRNA compared to scrambled-miRNA injected mice.

These in vivo results strongly support the key role of CD147 in metastasis formation by regulating the lymphangiogenic phenotype in melanoma.

## 4. Discussion

Recent studies have suggested that targeting lymphatic vessel sprouting through VEGFR-3 inhibition could be a potential therapeutic approach to melanoma treatment. In this context, the FLT4 antagonist, MAZ51, significantly reduced peri- and intratumoral melanoma cells’ proliferation by decreasing the number of lymphatic vessels and capillaries into mice lungs with induced melanoma [42]. Moreover, a reciprocal interaction through a paracrine mechanism between LECs and melanoma cells was recently shown by Shields et al., promoting melanoma metastasis [43]. Recently, Ubellacker et al. showed that melanoma cells are protected by lymph from oxidative stress and ferroptosis, and form more metastases than in the blood. Thus, the lymphatic environment increases metastatic melanoma cells’ survival [44]. Our study, demonstrating a role of CD147 in the lymphangiogenesis process, suggests it can represent a promising target for the inhibition of lymphatic vessels for the spread of metastatic melanoma.

Most tumor cells were shown to overexpress CD147 on their cell membrane [45], which is thought to interact in a paracrine manner with the tumor microenvironment and activate its cells, such as the stromal fibroblast, which becomes myofibroblasts, expressing more MMPs and facilitating tumor cell invasion [22,46,47]. In a similar paracrine manner, CD147 was shown to interact with endothelial cells to promote angiogenesis [48]. Indeed, we have already shown that CD147 promotes angiogenesis through VEGFR-2 regulation, increasing migration and tube formation in endothelial cells and tumor growth in vivo [20,21]. Since VEGFR-2 is expressed in both endothelial and lymphendothelial cells, we question whether CD147 could be involved in the regulation of the lymphangiogenesis process.

**Figure 7:**
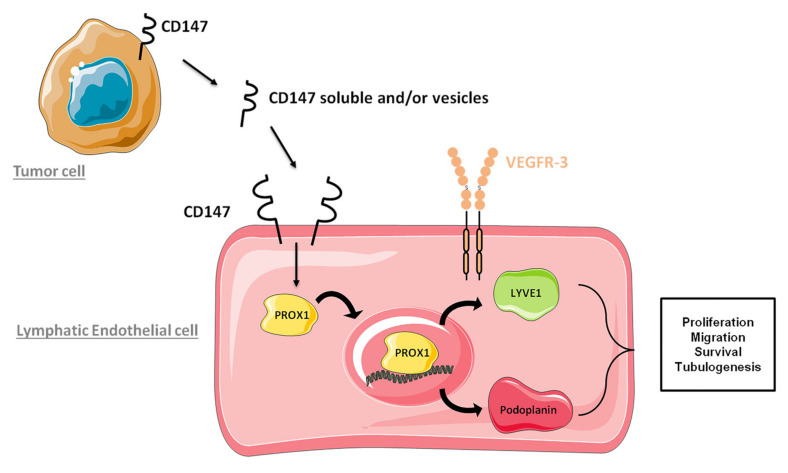
Schematic representation of paracrine CD147 effect on lymphangiogenesis regulation.

Here, we demonstrate that paracrine CD147 can also interact with the lymphatic cells and stimulate them to form lymphatic vessels expressing LYVE-1, Podoplanin, and VEGFR-3 (Figure 7).

The paracrine role of CD147 has already been demonstrated to occur through either its soluble form [47,49,50] and/or its membrane-vesicle-bound form [48,51,52], as both were shown to be secreted from tumor cells. In this study, we have shown that both the recombinant form that mimics the soluble form and the membrane form have similar paracrine effects on LEC migration and proliferation. However, it is difficult at this point to speculate as to which form of CD147 acts in vivo in our system.

When examining human melanoma lymph nodes, we found that high CD147 expression levels were correlated with a greater number of lymphatic vessels expressing these lymphatic-specific factors. These results confirm the role of CD147 in melanoma metastasizing through lymphangiogenic markers. Hence, beyond its established role in tumor growth, CD147 appears to promote tumor cell dissemination through the lymphatic system by stimulation of lymphendothelial cells, as demonstrated by its ability to increase the proliferation, migration, survival, and tubulogenesis of these lymphatic cells. These results were corroborated by the significant upregulation of lymphangiogenic factors’ expression at a transcriptional and translational level and the activation of their downstream signaling pathways. These results are in accordance with recent studies reporting that CD147 expression levels were significantly associated with nodal status in head and neck squamous cell carcinoma (HNSCC) [53], with cervical lymph node metastasis and invasion depth in cervical cancer [25] and with squamous cell carcinoma of the tongue at invasive stage, increasing the risk of cervical lymph node metastasis [54].

Importantly, we show that the regulation of lymphangiogenesis properties by CD147 is mediated by PROX-1 transcription factor, as rhCD147 treatment increased PROX-1 recruitment to lymphangiogenic targets promoters while PROX-1 silencing abrogated these observed effects. PROX-1 was shown to be necessary for the differentiation of lymphatic endothelial cells [11,55], and its expression was also correlated with lymphatic vascular invasion and metastasis in several types of cancers including esophagus squamous cell carcinoma (ESCC), colorectal cancer, and hepatocellular carcinoma [56,57,58]. Thus, PROX-1 seems to be involved in tumor progression by promoting cancer cell invasion and migration, but also in tumor metastasis through lymphangiogenesis regulation [59].

Despite promising preclinical results, anti-angiogenic therapies have had only limited clinical benefit; this could be due to primary or acquired resistance through the activation of alternative pro-angiogenic mechanisms. Even after treatment with anti-angiogenic agents, such as anti-VEGFR-2 antibodies, cancer patients remain vulnerable to lymphatic-vessel-mediated metastasis. This limitation of anti-angiogenic therapies could be explained by the hypoxic condition in tumors caused by these molecules as a hypoxic microenvironment is known to drive metastatic dissemination and resistance to therapy [60], but also by overexpression of lymphangiogenic factors, enhancing tumor metastasis through the lymphatic vessels [61,62]. Thus, lymphatic metastasis seems to be an alternative way of promoting tumor resistance to current anti-angiogenic therapies. To our knowledge, there are no FDA-approved anti-lymphangiogenic drugs, although a recent Phase I dose-escalation study evaluating a human monoclonal antibody LY3022856/IMC-3C5, specifically targeting VEGFR-3 in solid tumors or in advanced colorectal cancers, was investigated but showed insignificant antitumor activity because of a limited observed efficacy on tumor growth with a median PFS of only 6.3 weeks [63]. Thus, targeting established metastatic lesions through VEGFR-3-specific inhibition was not sufficient to overcome tumor dissemination [63,64,65]. Recently, Anlotinib, a novel anti-angiogenic multitarget tyrosine kinase inhibitor, led to improvement in PFS and OS for patients with NSCLC when used as a third-line treatment [66]. Moreover, a recent study reported that Anlotinib decreases new metastatic lesions in patients with advanced lung adenocarcinoma through lymphangiogenesis inhibition via VEGFR-3 signaling pathway targeting [67]. Thus, Anlotinib tumor metastasis inhibition’s efficacy is dependent on angiogenesis and lymphangiogenesis, and targeting multiple pathways seems to be more efficient as a therapeutic approach to cancer [68]. Since CD147 regulates both angiogenesis and lymphangiogenesis, its inhibition represents a potentially efficient approach, simultaneously inhibiting both processes in tumor dissemination [21,48].

## 5. Conclusions

Our findings provide evidence for the paracrine role of CD147 in the lymphangiogenesis process, suggesting that CD147 could be a promising target for the inhibition of melanoma-associated lymphangiogenesis.

## Data Availability

The datasets generated during and/or analyzed during the current study are available from the corresponding author on reasonable request.

## Supplementary Materials

The following are available online at https://www.mdpi.com/article/10.3390/cancers13194859/s1: Figure S1: Original (uncropped) Western Blot, Figure S2: CHO-CD147 modulates LECs properties, Figure S3: FLT4 expression in LECs treated with growth factors, Figure S4: In vivo metastatic index, Table S1: Human melanoma lymph nodes; patients’ main clinical data, Table S2: DNA ChIP qPCR gene information.
