# Supplementary material for: CD147 Promotes Tumor Lymphangiogenesis in Melanoma via PROX-1
Source: Cancers (Basel). 2021 Sep 28;13(19):4859. doi: 10.3390/cancers13194859 (PMC8508014; doi:10.3390/cancers13194859)
Supplement: Supplementary file 1 [file cancers-13-04859-s001.zip › Supplementary files - revision 2/Reger de Moura et al. - Supplementary Figures legends.pdf]

CD147 promotes tumor lymphangiogenesis in melanoma via PROX-1

Supplementary Figure S1. CHO-CD147 modulates LECs' properties. LECs were treated with or without CHO-CD147 and evaluated for effects on cell migration (a) and proliferation (b). Columns indicate the means of three independent experiments carried out in triplicate and bars indicate the SD; ***$p < 0.0001$.

Supplementary Figure S2. *FLT4* expression in LECs treatedwith growth factors. qRT-PCR of *FLT4* expression in LECs treated or not with a positive control mix (VEGF-A + bFGF + VEGF-C), using *PPIA* as a reference. Columns indicate the means of three independent experiments carried out in triplicate; bars indicate SD; ***$p < 0.0001$.

Supplementary Figure S3. *In vivo* metastatic index. Columns indicate the means of three independent experiments carried out in triplicate; bars indicate SD; ***$p < 0.0001$.
