# Supplementary material for: CD147 Promotes Tumor Lymphangiogenesis in Melanoma via PROX-1
Source: Cancers (Basel). 2021 Sep 28;13(19):4859. doi: 10.3390/cancers13194859 (PMC8508014; doi:10.3390/cancers13194859)
Supplement: Supplementary file 1 [file cancers-13-04859-s001.zip › Supplementary files - revision 2/Reger de Moura et al. - Supplementary Figures revision 2.pptx]

## Slide 1
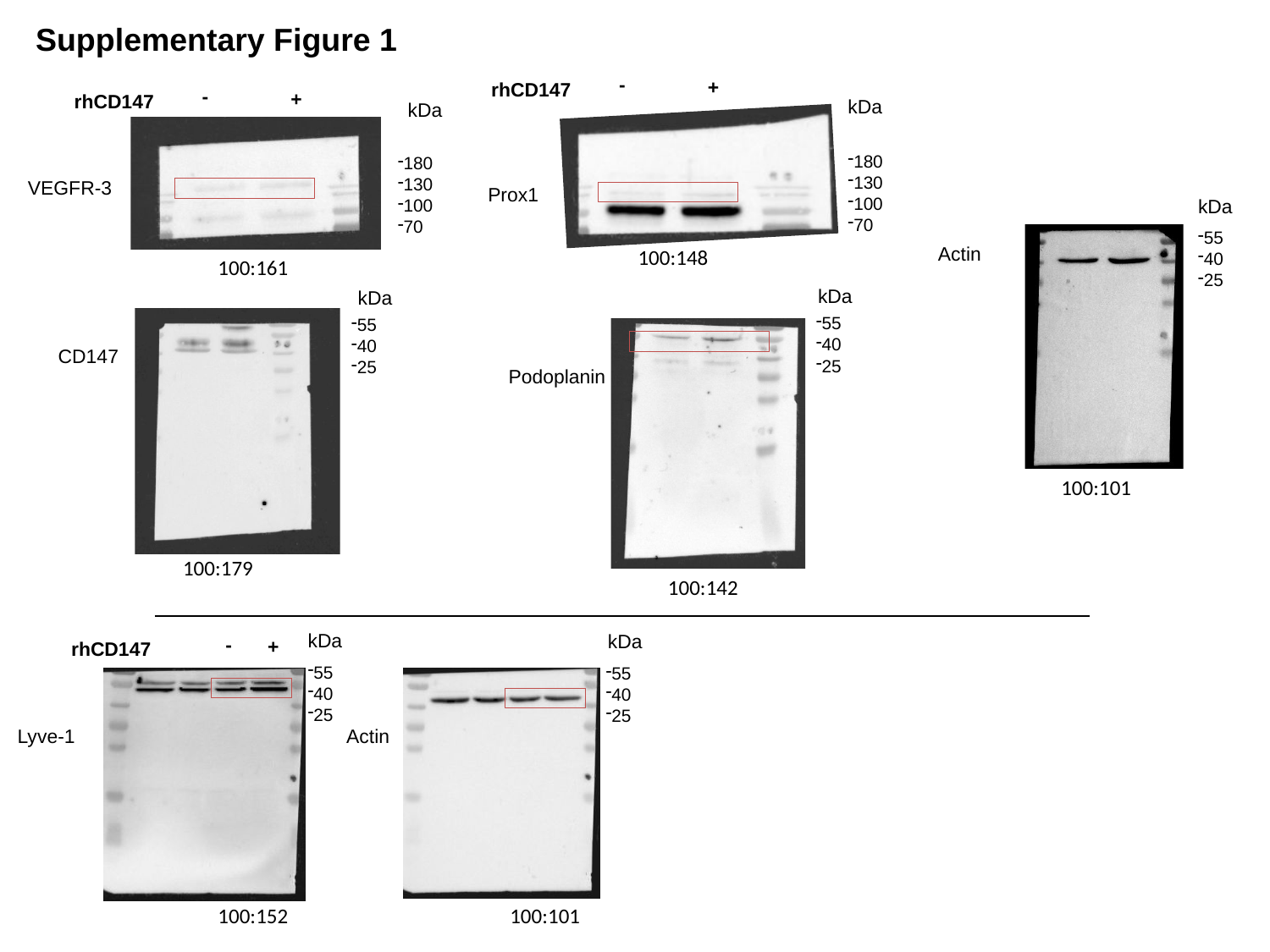

Supplementary Figure 1
-
+
rhCD147
-
+
rhCD147
kDa
kDa
180
130
100
70
VEGFR-3
Prox1
kDa
Actin
100:148
100:161
kDa
kDa
55
40
25
CD147
Podoplanin
100:101
100:179
180
130
100
70
55
40
25
55
40
25
100:142
kDa
kDa
-
+
rhCD147
Lyve-1
Actin
100:152
100:101
55
40
25
55
40
25

## Slide 2
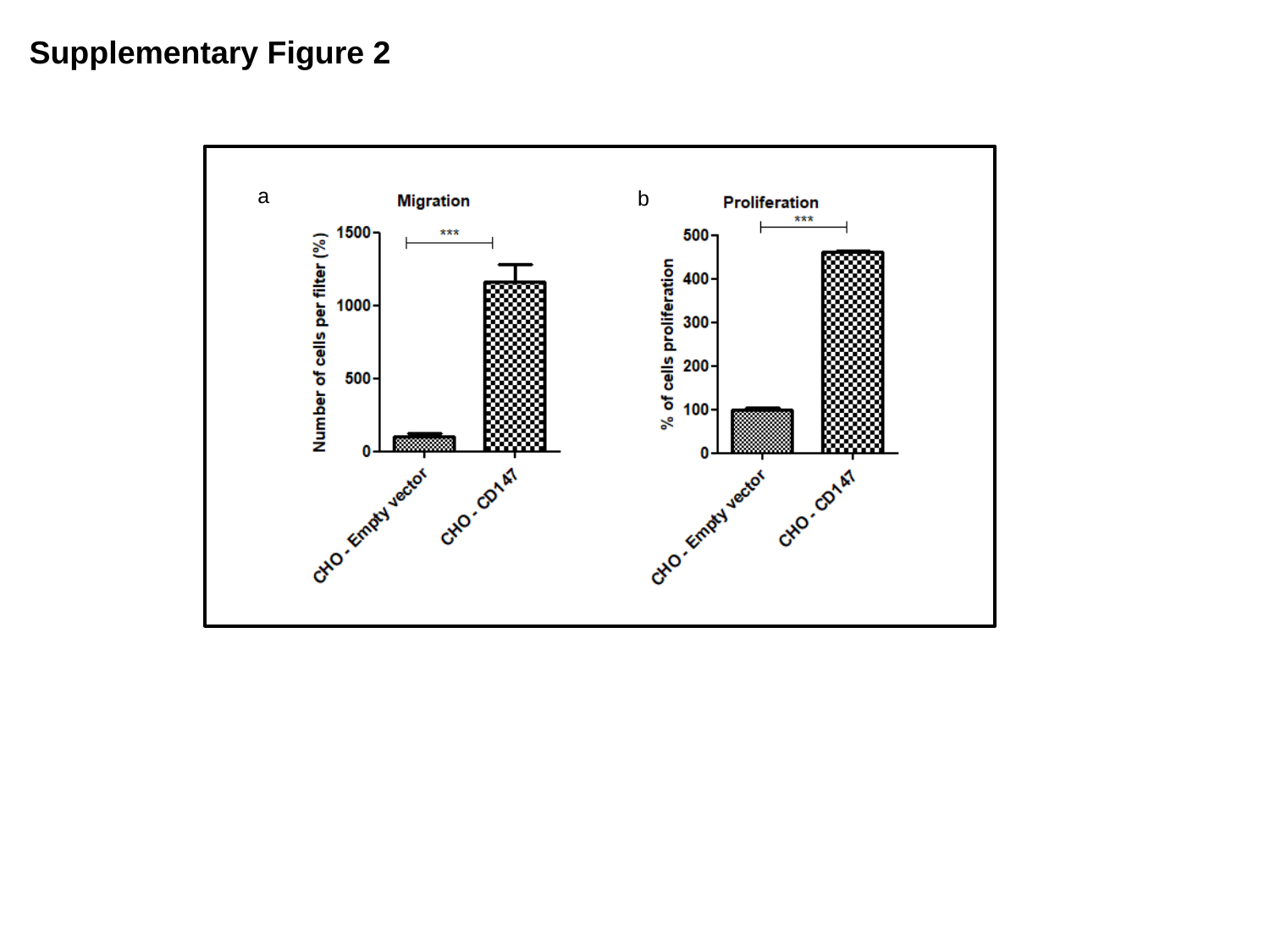

Supplementary Figure 2
a
b

## Slide 3
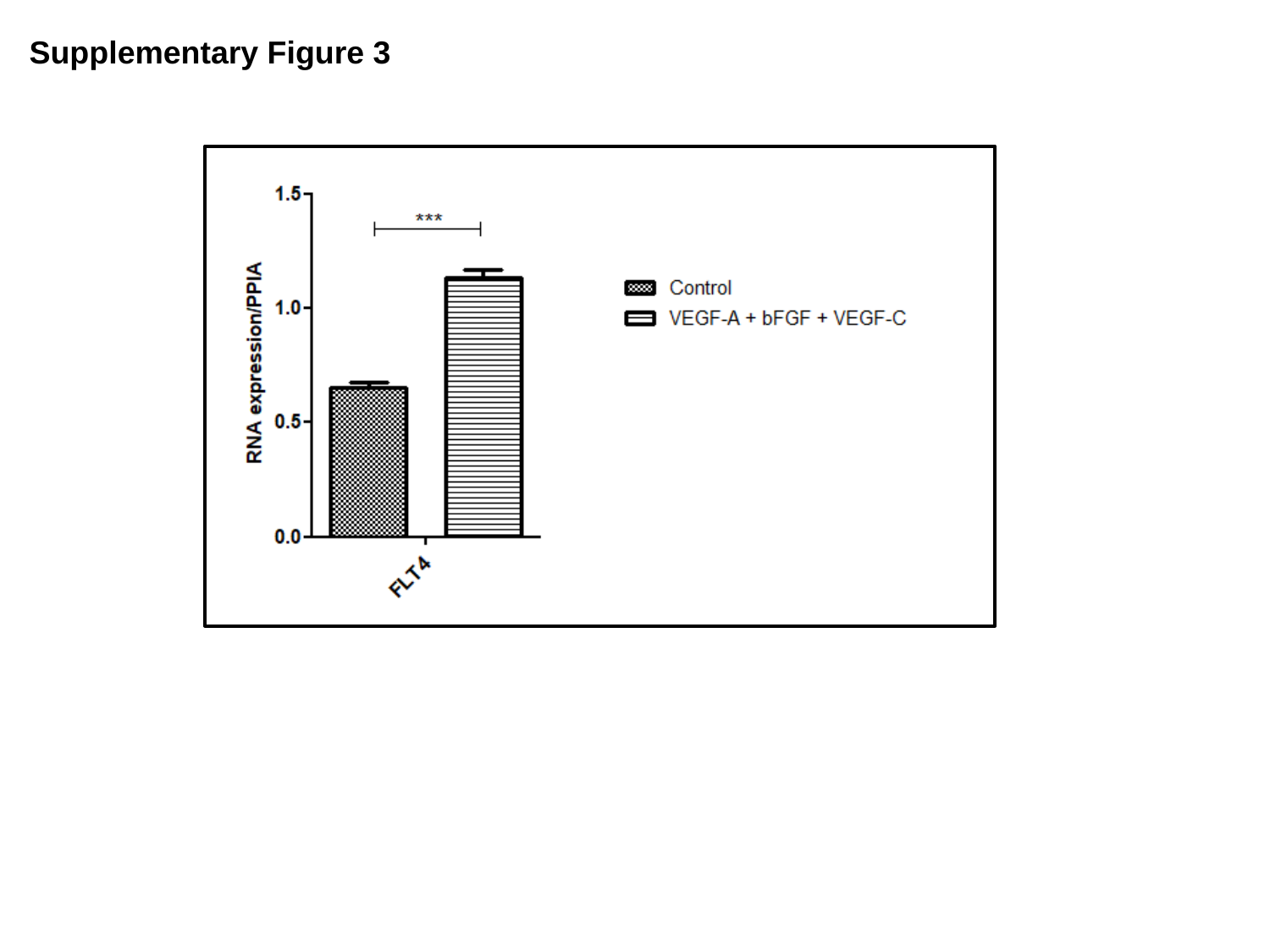

Supplementary Figure 3

## Slide 4
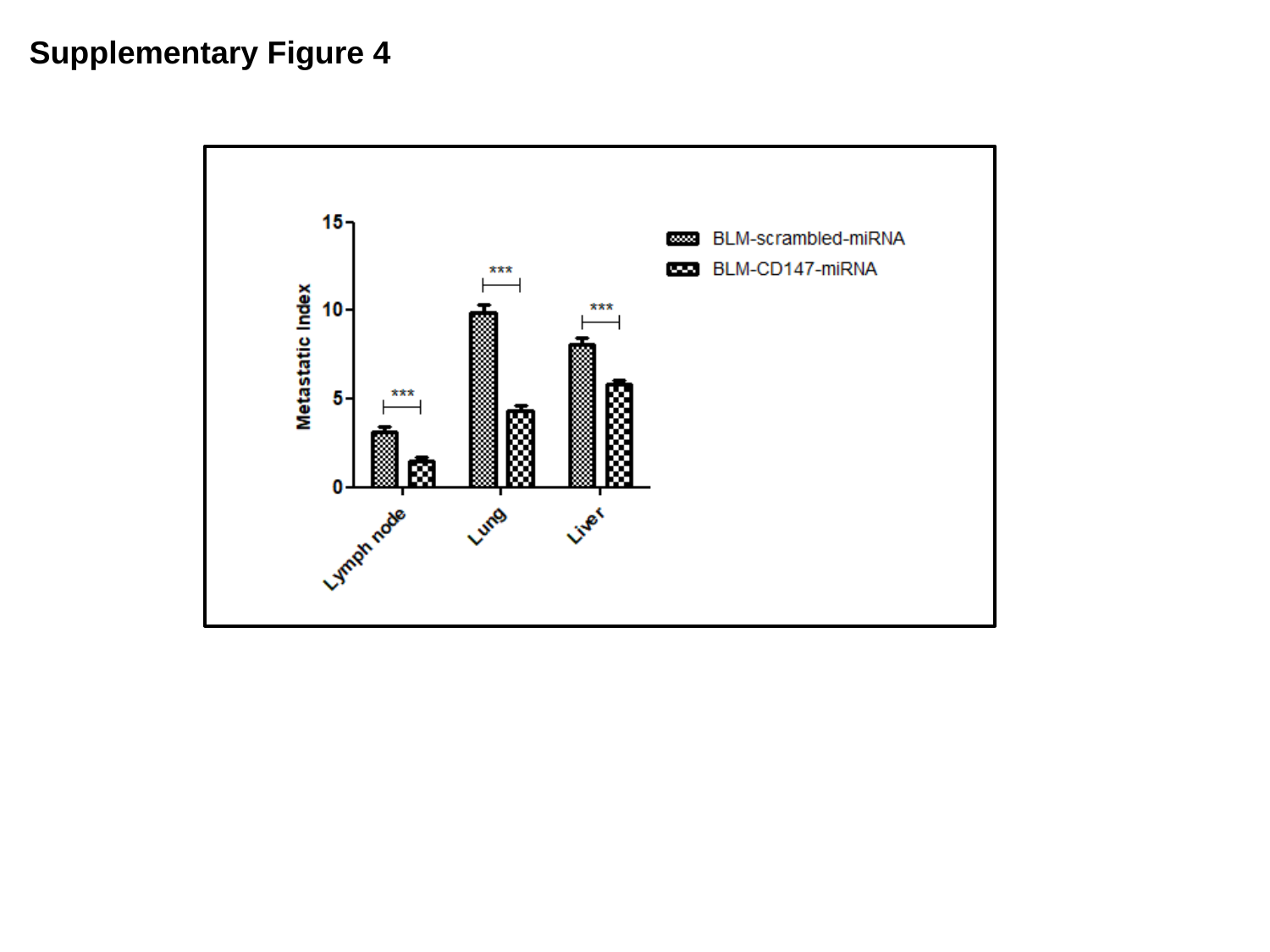

Supplementary Figure 4
